# Idiopathic Renal Infarction Mimicking Appendicitis

**DOI:** 10.1155/2017/8087315

**Published:** 2017-01-22

**Authors:** Marco Di Serafino, Rosa Severino, Chiara Gullotto, Francesco Lisanti, Enrico Scarano

**Affiliations:** ^1^Radiology Department, San Carlo Hospital, Potenza, Italy; ^2^Emergency Department, Cisanello Hospital, Pisa, Italy; ^3^Emergency Department, San Carlo Hospital, Potenza, Italy

## Abstract

Renal infarction is a rare cause of referral to the emergency department, with very low estimated incidence (0.004%–0.007%). Usually, it manifests in patients aged 60–70 with risk factors for thromboembolism, mostly related to heart disease, atrial fibrillation in particular. We report a case of idiopathic segmental renal infarction in a 38-year-old patient, presenting with acute abdominal pain with no previous known history or risk factors for thromboembolic diseases. Because of its aspecific clinical presentation, this condition can mimic more frequent pathologies including pyelonephritis, nephrolithiasis, or as in our case appendicitis. Here we highlight the extremely ambiguous presentation of renal infarct and the importance for clinicians to be aware of this condition, particularly in patients without clear risk factors, as it usually has a good prognosis after appropriate anticoagulant therapy.

## 1. Introduction

Renal infarction is an uncommon and easily overlooked condition. The reported incidence is very low varying between 0.004% and 0.007%, and because of its nonspecific presentation, the diagnosis is usually delayed from days to weeks after the onset of symptoms [1–3].

It can be differentiated into total and segmental renal infarction by using different imaging studies. There might be a significant renal function loss if the main renal artery is involved, whereas the clinical impact of segmental renal infarction on renal function is not well understood. Renal infarction usually occurs between the 6th and the 8th decade of life and the major causes are cardiac diseases like atrial fibrillation, myocardial infarction, and rheumatic mitral stenosis [4]. Other pathologies potentially involved are prosthetic valves, atrial or ventricular thrombi, arteriosclerosis, polyarteritis nodosa, lupus erythematous, trauma, and polycythemia vera [3, 5]. In addition, patients without underlying disease are also reported in the literature [6–8]. Renal infarction is variably associated with nonspecific symptoms like flank or abdominal pain, nausea, vomiting, fever, and hematuria, which could mimic a lot of different diseases [2, 3]. As a consequence, a late diagnosis of renal infarction is quite frequent in the emergency department and therefore with potential severe outcome [9]. Hence, it is crucial for clinicians and radiologists to be aware of this uncommon cause of flank/abdominal pain to avoid misdiagnosis and wrong therapeutic approaches.

## 2. Case Report

**Figure 1:**
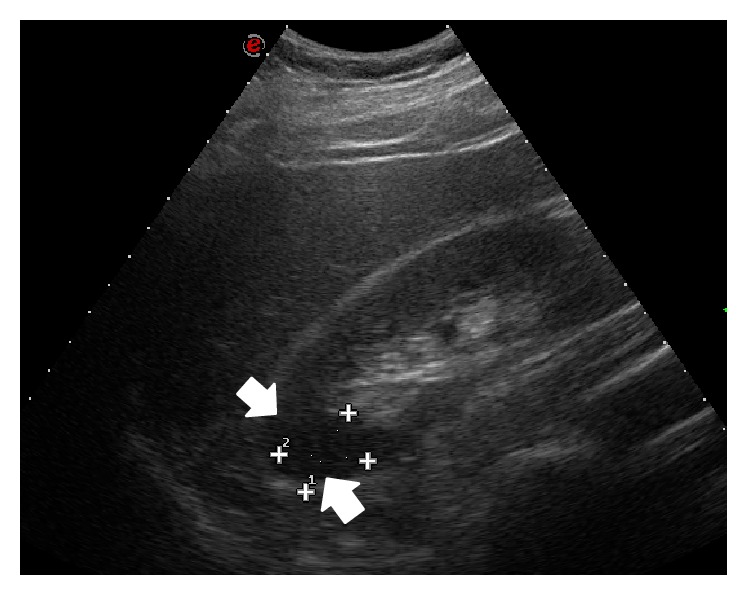
Ultrasonography of the abdomen with convex probe shows at the level of the middle-upper pole of the right kidney a wedge-shaped hypoechoic area, which appears clearly demarcated (arrows).

**Figure 2:**
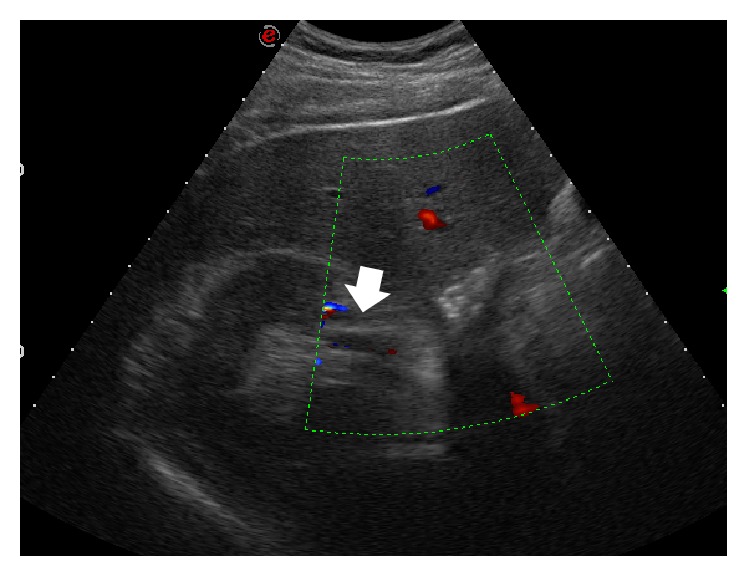
Ultrasonography of the abdomen with convex probe, transverse scan, color-Doppler study: at the level of the right kidney, there is lack of flow involving the median branch of a triple renal artery (arrow).

**Figure 3:**
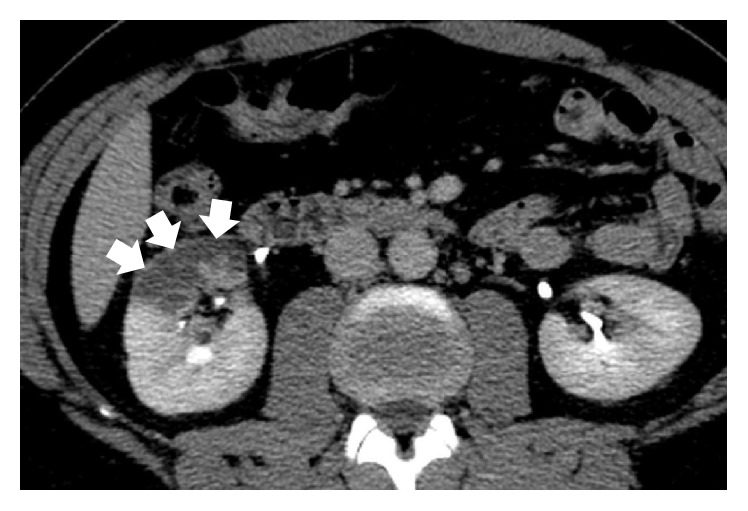
Contrast-enhanced CT scan, axial image, portal phase shows a cuneiform hypodense and nonenhancing area of 5 cm in diameter at the middle-upper pole of the right kidney (arrows).

**Figure 4:**
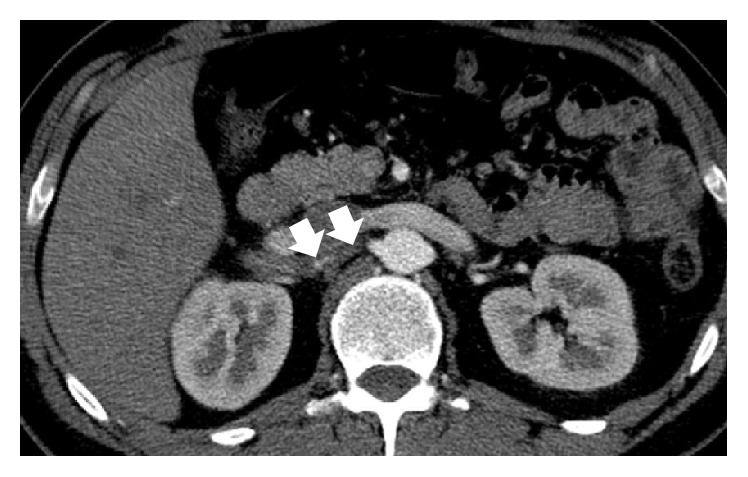
Contrast-enhanced CT scan, axial image, arterial phase shows intraluminal subtotal filling defect due to a thrombosis of a branch of the triple renal artery (arrows) from the origin up to its most peripheral ramifications.

A 38-year-old man with no significant past medical history was admitted to the emergency department presenting with sudden onset of discontinuous severe abdominal pain that was focused in the right quadrants and radiated to the right iliac fossa and to the back. The patient did not report any relief with common analgesic drugs (paracetamol, 1000 mg). Blood pressure and heart rate were within normal limits, 130/85 mm/Hg and 90 bpm, respectively. ECG showed normal sinus rhythm and no cardiac abnormalities. Physical examination found deep tenderness at McBurney's point and abdominal rebound tenderness, while there was not tenderness when eliciting the costovertebral angle. Laboratory tests revealed leukocytosis (12.000/*μ*L) and high levels of LDH (651 U/L), whereas kidney function tests and urine analysis did not show any significant alterations. Clinical and biological findings were initially suggestive for appendicitis and according to our internal guidelines an Ultrasound (US) was made to confirm the diagnostic hypothesis. The US examination was performed at first with a high-frequency probe (10–15 MHz) to evaluate the appendix and the surrounding area; however, no ultrasonographic abnormalities suggestive for appendicitis were detected. The US study was completed with a convex probe (5–7 MHz) to exclude other possible causes of abdominal pain and it showed, at the level of the middle-upper pole of the right kidney, a wedge-shaped hypoechoic area (Figure 1), which did not show any vascular signal at color and power-Doppler study, unlike the adjacent parenchyma. In addition, there was a triple renal artery with lack of perfusion of the median branch (Figure 2). The described hypoechoic area appeared clearly demarcated and suggestive for acute renal infarction. However, further diagnostic images were needed. A contrast-enhanced computed tomography (CT) was performed and showed a cuneiform low attenuation area of 5 cm in diameter at the middle-upper pole of the kidney (Figure 3). Moreover, there was a triple right renal artery, with an incomplete filling defect due to a thrombosis involving the median artery from the origin up to its most peripheral ramifications (Figure 4). This abnormality was likely related to the described hypovascular area of the renal parenchyma, with normal enhancement in the medium-caudal portion of the kidney (Figure 3). Beside continuous intravenous hydration, anticoagulation with enoxaparin was started immediately after the contrast-enhanced CT. Because of lack of identifiable causes, clotting studies including INR, antithrombin III, C3c, C4, and anticardiolipin antibodies were sent and came back negative. Thus, without other possible risk factors of thromboembolic disease, the patient was diagnosed with idiopathic acute renal infarction. Having had the patient's symptoms improved he was discharged home on warfarin. At one-year follow-up he had no clinical and US recurrence.

## 3. Discussion

Renal infarction is an uncommon and easily misdiagnosed condition. Characteristic clinical findings in major acute renal infarction include sudden onset of abdominal or flank pain, sometimes fever, and nausea with vomiting. Laboratory findings include moderate leukocytosis, albuminaemia, microscopic hematuria, and rising of LDH levels, which was demonstrated to be particularly prominent in renal infarction [1–3, 6]. These clinical and laboratory findings, although consistent with renal infarction, are nonspecific and often suggestive for alternative conditions including acute surgical abdomen or appendicitis with the risk of overlooking the correct diagnosis [6, 10]. Actually, other authors describe cases of not promptly recognized renal infarct [11, 12]. In particular, Xu et al. initially misinterpreted the presence of right flank pain with leukocytosis as appendicitis instead of renal infarct, as it happened in our case [11]. In addition, renal infarction is usually related to cardiac diseases, especially atrial fibrillation or other conditions of increased risk of thromboembolic episodes [2, 8]. However, Bolderman et al. [8] reported that there are several idiopathic cases where there are not clear risk factors related to renal infarct. Likewise in the case we presented, the patient was young and not affected by any predisposing conditions or previous thromboembolic events. This made the correct diagnosis more difficult. Therefore, imaging tools are important to make proper clinical assessment. The first diagnostic work-up of patients with acute abdominal pain includes US, which is widely available real-time dynamic exploration [13]. Moreover, the use of Doppler technique to evaluate the blood flow is an essential component of US study, particularly when the possibility of vascular impairment exists [14]. In our case, US study of the abdomen did not give any significant information; on the contrary, Doppler evaluation allowed achieving the correct diagnosis of renal infarction. Acute renal infarction appears as the absence of perfusion on color- and power-Doppler examination, complete when the entire kidney is affected or patchy when segmental arteries are involved. Absence of flow may also be directly appreciated in the renal artery or, in rare cases of venous thrombosis causing infarction, the renal vein [15, 16]. After a first hypothesis of renal vascular impairment is made at US, a contrast-enhanced CT scan is due to confirm the diagnosis. Actually, CT has become the gold standard for the diagnosis of renal infarction. In addition, it may assess if there is also evidence of ischemia in other organs such as spleen, liver, or lungs or whether there are indirect signs of cardiac disease responsible for thromboembolism episodes. Renal infarction usually appears at CT as wedge-shaped cuneiform area of low attenuation without contrast enhancement, within an otherwise normal appearing kidney [3]. The possibility of detecting renal arterial infarction with US and CT scan should minimize the necessity of invasive procedures, such as retrograde pyelography or renal arteriography, and help in making a quick differential diagnosis with other more common acute abdominal diseases such as appendicitis [17–19]. As a consequence, an appropriate treatment can be started, which is the same for both idiopathic cases and patients with atrial fibrillation-induced embolism and is based on early anticoagulation (low molecular weight heparin) with a good prognosis [2, 8, 11]. Other possible treatments include endovascular thrombolysis, which is, however, supported by limited data so that its utility is still debated [11, 20].

## 4. Conclusion

In conclusion, even though nonspecific clinical symptoms and laboratory findings may suggest a more frequent cause of acute abdomen such as appendicitis, clinicians and radiologists should also think about renal infarction, even in young patients without structural or arrhythmic cardiac disease. US exam and contrast-enhanced CT scan may help in the differential diagnosis, so that a fast intervention can avoid the progression of the infarction and improve the patient's prognosis.
